# The complete mitochondrial genome of *Gymnogobius Petschiliensis* (Gobiiformes; Gobiidae; Gobionellinae) and its phylogenetic implications

**DOI:** 10.1080/23802359.2017.1398609

**Published:** 2017-11-21

**Authors:** Li Gong, Wei Chen, Li-Qin Liu, Zhen-Ming Lü

**Affiliations:** a National Engineering Laboratory of Marine Germplasm Resources Exploration and Utilization, Zhejiang Ocean University, Zhoushan, China;; b National Engineering Research Center for Facilitated Marine Aquaculture, Marine Science and Technology College, Zhejiang Ocean University, Zhoushan, China

**Keywords:** Floating goby, mitogenome, phylogenetic evolution

## Abstract

Of the goby fishes, many *Gymnogobius* species have been poor recognized mainly because of the absence of enough molecular information and clear phylogenetic framework. In this study, the complete mitochondrial genome of *Gymnogobius petschiliensis* was determined and described. The mitogenome is 16,422 bp in length and consists of 22 tRNAs, 13 protein-coding genes, two rRNAs, one control region and a light strand replication origin (O_L_). The arrangement of this mitogenome is identical to that of the typical teleost. The overall base composition is 27.5%, 29.5%, 26.1%, and 16.9%, for A, T, C, and G, respectively, with a slight bias on A + T content (57.0%). The 13 protein-coding genes use the initiation codon ATG except *COI*, which uses GTG. Most of them use TAA or TAG as the stop codon, while *COII*, *COIII*, and *Cyt b* use an incomplete T or TA and *ND4* uses an unusual AGA. The maximum-likelihood phylogeny tree of 19 Gobionellinae species demonstrated that *G. petschiliensis* had a very closely relationship with the same genus *G. urotaenia*. This study is expected to contributing to the phylogenetic evolution of *G. petschiliensis* and further phylogenetic relationship of Gobionellinae and Gobiiformes.

Gobiidae is one of the largest families with about 210 genera (at least 1950 species) (Nelson 2006). However, there are many species have not been well described. Of the goby fishes, for instance, many *Gymnogobius* species have been poor recognized mainly because of the absence of enough molecular information and clear phylogenetic framework (Kim et al. 2004). Mitochondrial DNA, which has been proved useful in species identification and phylogenetic studies, has great potential to resolve this issue (Miya et al. 2003; Tornabene et al. 2013; Harrington et al. 2016). In the present study, we determined and described the complete mitogenome of *G. petschiliensis*, and reconstructed the phylogenetic relationship of the relative species of Gobionellinae, expecting for better understanding the systematic evolution of the genus *Gymnogobius* and further phylogenetic study of Gobionellinae.

The specimen was collected from Qingdao in Shangdong, China (36.0412°N; 120.1923°E) and was stored in 95% ethanol with accession number 20131115NA05. Eleven primers were designed to amplify the complete mitochondrial sequence according to previous species (Kong et al. 2009; Shi et al. 2011; Gong et al. 2017b). The total length is 16,422 bp (GenBank accession no. MG018480), including 22 tRNAs, 13 protein-coding genes, two rRNAs, one control region and a light strand replication origin (O_L_). Most of the genes are encoded by heavy strand, while *ND6* and other eight tRNAs are encoded by light strand. The gene arrangement of this species is identical to that of typical teleost (Boore 1999; Ponce et al. 2008; Gong et al. 2017a). The overall base composition is A 27.5%, T 29.5%, C 26.1%, G 16.9%, and A + T content 57.0%.

The 22 tRNA genes were interspersed between rRNA and protein-coding genes, with sizes ranging from 65 bp (*tRNA-Cys*) to 75 bp (*tRNA-Lys*). The 13 protein-coding genes use the initiation codon ATG except *COI*, which uses GTG. Most of them use TAA or TAG as the stop codon, while *COII*, *COIII*, and *Cyt b* use an incomplete T or TA and *ND4* uses an unusual AGA. The 13 protein-coding genes encode 3810 amino acids in total. The two rRNA genes (12S and 16S) are generally isolated by *tRNA*-*Val*, located between *tRNA*-*Phe* and *tRNA*-*Leu*. The 48 bp O_L_ is located between the *tRNA-Asn* and *tRNA-Cys* genes in a cluster of five tRNA genes (WANCY region). The 925 bp CR is commonly situated between *tRNA-Pro* and *tRNA-Phe* genes. The symbolic structures are observed as in other teleosts: termination-associated sequences (TAS), central conserved sequence blocks domain (CSB-F, D, B, A), and conserved sequence blocks (CSB-2, 3) (Guo et al. 2003; Xu et al. 2011; Gong et al. 2015).

In order to explore the systematic status of *G. petschiliensis* and further phylogenetic study of Gobionellinae, a maximum-likelihood tree was constructed based on the 13 protein-coding genes from 19 Gobionellinae species, with *Tridentiger obscurus* and *T. barbatus* as the outgroup. The tree clearly showed that all Gobionellinae species clustered into a group and *G. petschiliensis* formed a sister-group with *G. urotaenia*, suggesting a very closely relationship of these two species (Figure 1).

**Figure 1.**
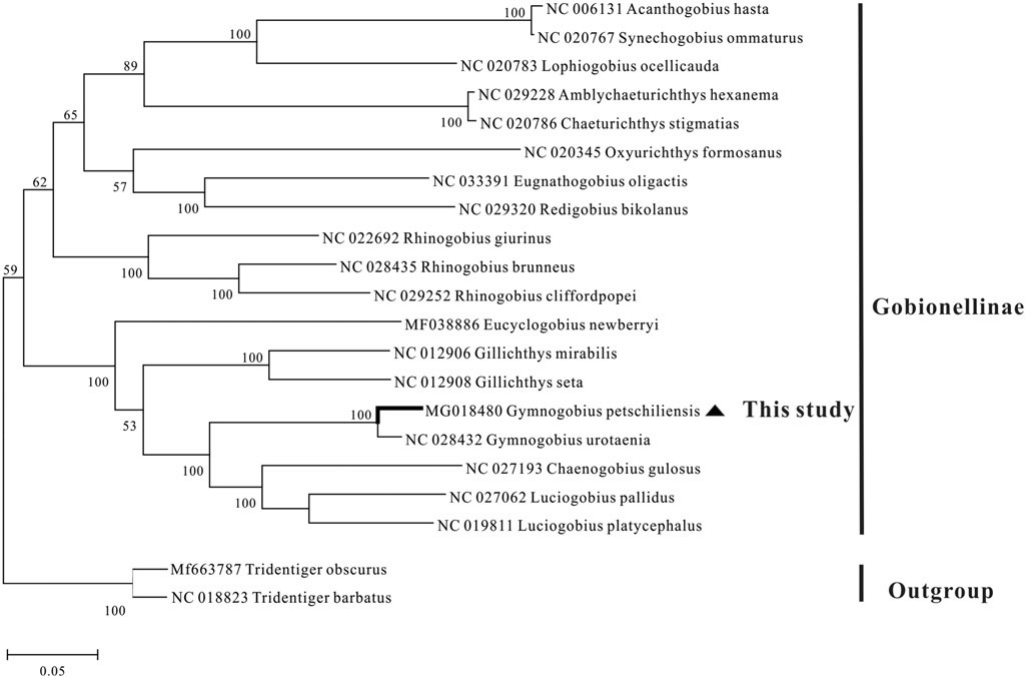
Phylogenetic tree of Gobionellinae based on the maximum-likelihood (ML) analysis of 13 protein-coding genes. The GTR + I + G model was the most appropriate model based on the Akaike Information Criterion (AIC). The number at each node is the bootstrap probability. The number before the species name is the GenBank accession number.
